# Time Evolution of Sublingual Microcirculatory Changes in Recreational Marathon Runners

**DOI:** 10.1155/2017/7120785

**Published:** 2017-07-30

**Authors:** Andrius Pranskunas, Justina Arstikyte, Zivile Pranskuniene, Jurga Bernatoniene, Inga Kiudulaite, Egle Vaitkaitiene, Dinas Vaitkaitis, Marius Brazaitis

**Affiliations:** ^1^Department of Intensive Care Medicine, Lithuanian University of Health Sciences, Kaunas, Lithuania; ^2^Institute for Digestive Research, Lithuanian University of Health Sciences, Kaunas, Lithuania; ^3^Department of Drug Technology and Social Pharmacy, Lithuanian University of Health Sciences, Kaunas, Lithuania; ^4^Department of Disaster Medicine, Lithuanian University of Health Sciences, Kaunas, Lithuania; ^5^Sports Science and Innovation Institute, Lithuanian Sports University, Kaunas, Lithuania

## Abstract

We aimed to evaluate changes in sublingual microcirculation induced by a marathon race. Thirteen healthy male controls and 13 male marathon runners volunteered for the study. We performed sublingual microcirculation, using a Cytocam-IDF device (Braedius Medical, Huizen, Netherlands), and systemic hemodynamic measurements four times: 24 hours prior to their participation in the Kaunas Marathon (distance: 41.2 km), directly after finishing the marathon, 24 hours after the marathon, and one week after the marathon. The marathon runners exhibited a higher functional capillary density (FCD) and total vascular density of small vessels at the first visit compared with the controls. Overall, we did not find any changes in sublingual microcirculation of the marathon runners at any of the other visits. However, in a subgroup of marathon runners with a decreased FCD compared to the subgroup with increased FCD, the subgroup with decreased FCD had shorter running time (190.37 ± 30.2 versus 221.80 ± 23.4 min, *p* = 0.045), ingested less fluids (907 ± 615 versus 1950 ± 488 mL, *p* = 0.007) during the race, and lost much more weight (−2.4 ± 1.3 versus −1.0 ± 0.8 kg, *p* = 0.041). Recreational marathon running is not associated with an alteration of sublingual microcirculation. However, faster running and dehydration may be crucial for further impairing microcirculation.

## 1. Introduction

Evidence suggests that exercise training and physical activity have health benefits and reduce cardiovascular disease risks [1]. However, prolonged intense running transiently elevates the risk of cardiac events [2] and the probability of sudden cardiac death even of young individuals [3]. Also, during long-distance run, various gastrointestinal symptoms may occur ranging from mild nausea to hemorrhagic stool. Incidence rates of such symptoms vary from 36 to 71% [4] and in some cases continue during the first 24 h after the run [5]. Although the etiology of intense exercise related to gastrointestinal symptoms is thought to be multifactorial, splanchnic hypoperfusion has been postulated as a key mechanism including healthy young individuals [6].

Microcirculatory perfusion of sublingual mucosa is a part of interest because it is easy and noninvasively accessible, its changes are related to mortality, and it is a part of the upper digestive tract. Sublingual microcirculation has been considered as a surrogate measure for gut mucosal microcirculation because the lingual region is an embryonic derivate of the digestive tract and correlates with sublingual capnometry and gastric tonometry in spite of different vascularization [7]. However, sublingual microcirculation sometimes does not correlate with intestinal microcirculation, especially in sepsis [8, 9].

Microcirculatory imaging techniques, such as sidestream dark field (SDF) imaging and new generation incident dark field (IDF) imaging, have enabled the direct observation of microcirculation at the bedside [10, 11]. These techniques visualize blood flow at the level of individual capillaries in available thin mucosa (often sublingual) and help to assess capillary flow and density in humans. Clinical studies have shown that alterations of sublingual microcirculation, such as decreased functional capillary density (FCD) and flow, are associated with worse outcomes [12, 13]. Given the possible influence of marathon races on outcomes and digestive tract mucosal perfusion, we hypothesize that a marathon race decreases sublingual capillary perfusion.

This study aimed to evaluate acute changes in sublingual microcirculation induced by a single marathon race.

## 2. Materials and Methods

### 2.1. Subjects

Thirteen healthy male controls and 13 male recreational marathon runners with a training volume of at least 5 h per week for a minimum of 2 years volunteered for the study. The healthy control subjects were moderately physically active (<2 h·week^−1^), but they did not participate in any formal physical exercise or sports program. The exclusion criterion was any history of cardiopulmonary or gastrointestinal disease. Each subject read and signed a written informed consent form that was consistent with the principles outlined in the Declaration of Helsinki. The Ethics Committee of Lithuanian University of Health Sciences (number BEC-MF-30) approved this study.

### 2.2. Protocol

We performed microcirculatory and systemic hemodynamic measurements four times: 24 hours prior to their participation in the Kaunas Marathon (distance: 41.2 km), directly after finishing the marathon, again 24 hours after the marathon, and one week after the marathon. During these four visits, the same examinations were performed at the same time between 12:00 and 16:00; the subjects were in a nonfasting state in a quiet room with a stable temperature between 19°C and 22°C. After completing the full marathon, athletes arrived at the testing station within 15 min and were immediately tested. The measurements were performed with athletes lying with the upper part of their body at a 45° angle. We measured systemic hemodynamic parameters using impedance cardiography (Niccomo, Medis, Medizinische Messtechnik GmbH, Germany). Running time was measured using a professional timer by a trained person. The amount of fluids during and after running was measured by calculating the cups of ingested water.

Before starting the study, we measured participants' maximal oxygen uptake (VO_2_max). We measured VO_2_max using the ramp exercise test. This test consists of a 4-minute warm-up followed by an incremental continuous increase in speed of 0.1 km/h every 6 s until volitional fatigue. The criteria used to verify that VO_2_max was achieved included a respiratory exchange ratio greater than 1.1, a maximum heart rate (HR) equal to 220 – age ± 10 beats per min, and a plateau in oxygen uptake with increasing workload (all the criteria had to be met) [14]. We analyzed pulmonary gas exchange using a portable analyzer (Oxycon Mobile; Jaeger, Hoechberg, Germany). Prior to each test, the equipment was calibrated according to the manufacturer's recommendations.

### 2.3. Evaluation of Microcirculation

We obtained images of microcirculation from the sublingual mucosa using a Cytocam-IDF device (Braedius Medical, Huizen, Netherlands). This device is technically and optically optimized for the visualization of microcirculation on the surfaces of organs. IDF imaging is based on the principle that emitted green light (wavelength: 530 nm) is absorbed by the hemoglobin content in red blood cells [10]. Therefore, red blood cells are seen as black or gray bodies during imaging. The vessel walls are not visualized, so vessels can only be detected by the presence of red blood cells. Despite technological differences between the IDF device and the heavier SDF device (120 g versus 320 g), the pen-like Cytocam instrument provides a larger field of view (1.55 × 1.16 mm versus 0.9 × 1.0 mm). A recently published validation study demonstrated that Cytocam-IDF imaging detected more capillaries and yielded better image quality than SDF imaging [11]. The Cytocam device uses a sensor-based imaging approach to generate images versus the SDF device that uses video camera technology. The combination of better optics and resolution with a larger field of view (FOV) will logically yield more vascular observations in the FOV.

After gentle removal of saliva and other secretions using isotonic saline-drenched gauze, the Cytocam lens was applied to the sublingual region, avoiding pressure artifacts by establishing a threshold image. We first adjusted the optimal focus depth and contrast. Steady images of 6 seconds from at least three areas were acquired and stored on a computer. The image clips were exported for the analysis using validated AVA v3.0 software (MicroVision Medical, Amsterdam, Netherlands) [15]. Two investigators blindly analyzed the video clips offline in a random order to prevent coupling. Each image was divided into four equal quadrants. The flow was quantified by eye (no flow: 0; intermittent flow: 1; sluggish flow: 2; continuous flow: 3) per quadrant for each vessel diameter cohort (small: 10–20 *μ*m; medium: 21–50 *μ*m; large: 51–100 *μ*m). We calculated the microvascular flow index (MFI) as the sum of each quadrant score divided by the number of quadrants in which the vessel type was visible. The final MFI was averaged over a minimum of 12 quadrants (three regions, four quadrants per region) derived from the overall flow impressions of all vessels with a particular range of diameters in a given quadrant [16, 17].

We calculated the total vessel density (TVD) of small vessels using the AVA software package and a cut-off diameter for small vessels (mostly capillaries) of <20 *μ*m.

We defined the proportion of perfused vessels (PPV) of small vessels in terms of the percentage of crossings with perfused small vessels per total length of three equidistant horizontal and three equidistant vertical lines. We calculated FCD by multiplying the vessel density by the proportion of perfused vessels of small vessels. This method is described elsewhere by De Backer et al. and is in accordance with reports of a round-table conference [18].

We arbitrarily stratified the marathon runners into two groups (decreased FCD and increased FCD) according to the change in FCD from Visit I (prior to the race) to Visit II (after the race).

### 2.4. Statistics

Our primary outcomes were the sublingual FCD and TVD of small vessels. We used the Statistical Package for the Social Sciences (SPSS v15.1 for Windows, Chicago, IL, USA) for statistical analysis. We checked the data for normality using the Shapiro-Wilk test. Because no deviation from normality was detected, we report the data as mean ± standard deviation (SD). We analyzed these data using parametric tests. A one-way repeated-measures ANOVA model with a Bonferroni correction was used to compare multiple sets of related values [17]. For the subgroup statistical analysis, we performed nonparametric tests. We considered a *p* value of less than 0.05 to be statistically significant.

## 3. Results

The baseline characteristics of the study participants are presented in Table 1. There were no significant differences in age, body weight, or height between the two groups. The marathon runners had higher mean arterial pressure (MAP), systemic vascular resistance index (SVRI), maximum oxygen uptake (VO_2_max), FCD, and total vascular density (TVD) of small vessels at the first visit compared with the controls.

### 3.1. Changes in Systemic Hemodynamic Parameters over the Course of the Four Visits

**Figure 1:**
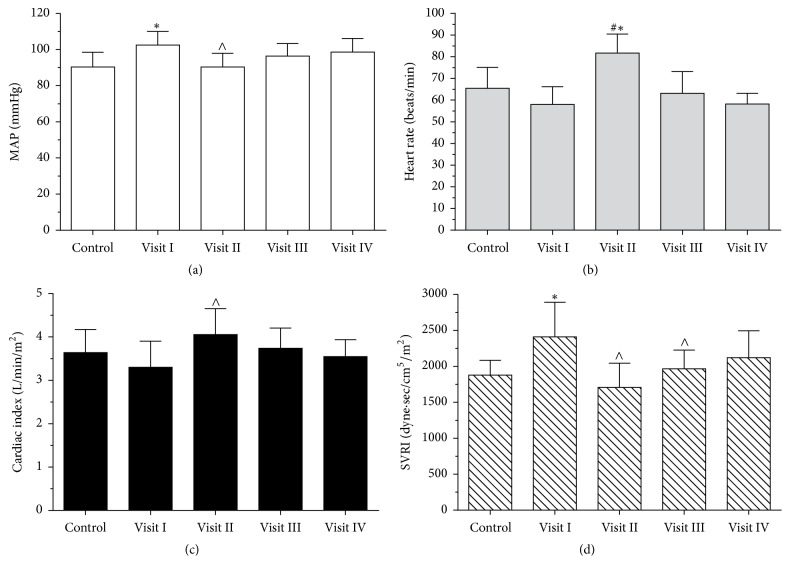
Changes in systemic hemodynamic parameters in marathon runners during the study period. Histograms of (a) mean arterial pressure,(b) heart rate, (c) cardiac index, and (d) systemic vascular resistance index. ^*∗*^*p* < 0.05 compared with controls; ^∧^*p* < 0.05 compared with Visit I; ^#^*p* < 0.05 compared with other visits.

We found that MAP and SVRI were significantly lower immediately after the marathon race (Visit II) compared with Visit I; heart rate (HR) and cardiac index (CI) were significantly higher (Figure 1). We found that HR during Visit II was significantly higher compared to all those of the other visits. During the week after the marathon, the parameters attained their baseline values, and no significant differences persisted between Visits III and IV. We found a strong correlation between the amount of fluids during running and running duration (*r* = 0.71, *p* = 0.007).

### 3.2. Changes in Microcirculation over the Course of the Four Visits

**Figure 2:**
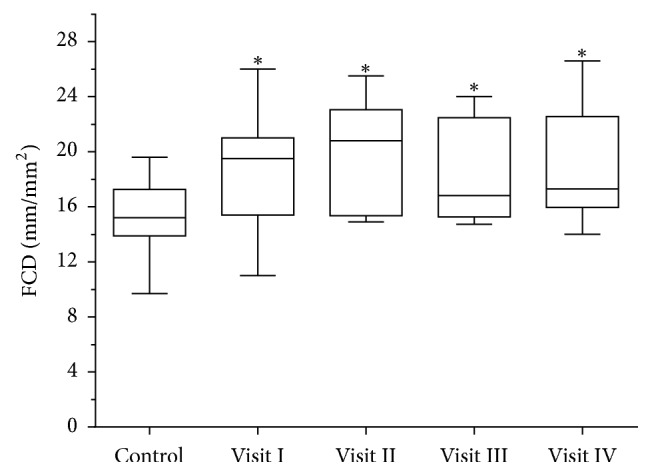
Box plots of sublingual functional capillary density (FCD) in marathon runners (Visits I–IV) and healthy control individuals. ^*∗*^*p* < 0.05 compared with controls.

**Figure 3:**
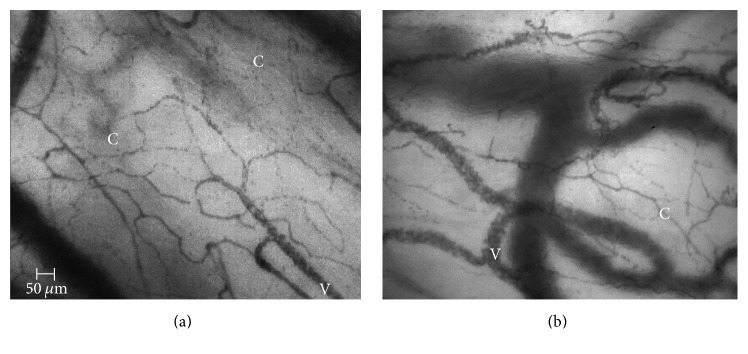
Digital microphotographs of microcirculation before ((a) FCD = 23.70 mm/mm^2^) and immediately after ((b) FCD = 20.32 mm/mm^2^) marathon race in marathon race winner. C: capillaries; V: venules.

Overall, we did not find any changes in sublingual microcirculation in marathon runners over the course of the four visits (Table 2, Figure 2). However, the group of marathon runners (*n* = 6) with the decreased FCD after finishing the race compared with the cohort with an increased FCD (*n* = 7) were characterized by a shorter race time, the ingestion of less fluids during the race, and larger weight loss (Table 3). Digital microphotographs of microcirculation before and immediately after marathon race in marathon race winner are presented in Figure 3.

## 4. Discussion

To the best of our knowledge, this study is the first to investigate the impact of marathon running on capillary perfusion of noninvasively accessible sublingual mucosa. Although our results did not reveal significant changes in sublingual microcirculation in marathon runners over the entire study period, we detected factors that can influence the alteration of microcirculation after a marathon race.

During intense exercise, the release of norepinephrine near the alfa-adrenoreceptors of the sympathetic nervous system induces splanchnic vasoconstriction, thereby increasing total splanchnic vascular resistance [19]. Thus, splanchnic blood flow may be decreased rapidly up to 80% through redistribution to provide sufficient blood flow to working muscle, heart, lungs, and skin [20]. At the same time, vascular resistance in working organs, such as the heart, lungs, active muscle, and skin, is decreased [21]. As blood is shunted from viscera to the active tissues, mucosal hypoperfusion and ischemia may result in the digestive tract, as well as increases in mucosal permeability [6, 22]. This may be a cause of digestive symptoms, such as nausea, vomiting, abdominal pain, or bloody diarrhea, during and after a marathon race [4, 5].

Gastric mucosal perfusion was evaluated by the investigators mostly using gastric tonometry [6, 21]. Gastric tonometry cannot represent perfusion at the capillary level and it is invasive. Also, due to technical problems, in particular duodenogastric reflux and feeding, it can interfere with PCO2 measurements, and thus it is now abandoned in practice. Sublingual evaluation using IDF imaging is noninvasive and represents perfusion at the capillary level.

Capillary perfusion, which refers to capillary flow and density, is the primary prerequisite for adequate tissue oxygenation and therefore the organ function [23]. Clinical studies have shown that alterations in sublingual microvascular perfusion are one of the strongest predictors of outcome and remain independently associated with outcome [12, 24]. We focused on runners with a decreased FCD after the race and found that race time and dehydration might be crucial factors for altering microcirculation during a marathon.

Studies have shown that hypovolemia in clinical settings may decrease capillary perfusion in the sublingual mucosa [25]. In our study, we found that runners in the decreased-FCD subgroup lost more fluids due to dehydration (mean: 2.4 kg of body weight), which might explain the alteration in their microcirculation.

Pressler and colleagues [26] used a nonmydriatic fundus camera to evaluate acute and chronic structural alterations of retinal vessel diameters in middle-aged, male marathon runners. These authors found that marathon runners had significantly larger arterioles and smaller venules compared with controls. Although the authors do not study capillaries, as we did in sublingual mucosa, they supplement information about possible arteriolar vasodilatation in the brain.

Compared with the controls, the marathon runners exhibited a higher FCD. This finding may be due to the fact that all of the capillaries were already perfused to prevent ischemic damage during a marathon race. Ischemic preconditioning-induced microcirculatory protection appears to be a systemic rather than a local phenomenon [27]. Ischemic preconditioning leads to the activation of nerve pathways and the release of biochemical angiogenic factors (e.g., VEGF), prevents endothelial dysfunction induced by ischemia-reperfusion injury, and supports higher NO production [28].

Marathon running is stressful exercise in which cardiac output, stroke volume, and HR are in a high steady state [29]. We found that HR and cardiac index after the race were significantly higher than those before the race. Evidence has shown that long-term endurance exercise results in enlarged arteries, decreased arterial wall thickness, and improved acetylcholine-mediated vasodilatation [30]. Furthermore, chronic adaptations are associated with a significant decrease in peripheral blood pressure, peripheral arterial resistance, and HR [31]. In strength-trained runners, the opposite effects may be observed (e.g., an increase in arterial stiffness and a slight increase in blood pressure) [32]. In our study, marathon runners had higher mean arterial pressure and systemic vascular resistance exploring the experience in marathon running. According to the literature, marathon runners have an increased coronary artery calcium score [2] and a higher prevalence of carotid and peripheral atherosclerosis [33].

We found some links between running speed and alteration of microcirculation. Two large studies have demonstrated that fast runners (i.e., those individuals running typically faster than 6 miles an hour) appear to gain no mortality benefit compared with nonrunners [34, 35]. In addition, strenuous jogging is not associated with better survival compared with sedentary nonjoggers.

Therefore, evaluating sublingual microcirculation to control the physiological balance between oxygen transport and consumption at the capillary level during marathon running may help identify the optimal individual running dose and intensity.

### 4.1. Study Limitations

We included only male participants in our study, and our results cannot readily be extrapolated to female runners. Furthermore, only two participants were included from the top 10 finishers of the marathon race; a more accurate assessment of leading recreational runners is necessary.

## 5. Conclusions

Recreational marathon running is not associated with an alteration of the sublingual microcirculation. Recreational marathon runners exhibited a higher FCD compared with age-matched controls, which indicates the beneficial effects of regular marathon running. However, faster running and dehydration may be crucial for further impairment of microcirculation.

## Figures and Tables

**Table 1:** Baseline characteristics of the study participants.

Variables	Marathon runners (*n* = 13)	Controls (*n* = 13)	*p*
Age (yr)	34 ± 7	29 ± 6.2	0.106
Mass (kg)	76.3 ± 5.3	82.0 ± 13.2	0.238
Height (cm)	181.9 ± 7.0	183.8 ± 5.4	0.291
Body mass index (kg/m^2^)	23.1 ± 2.0	24.2 ± 3.1	0.531
VO_2_max (mL·kg^−1^·min^−1^)	59.7 ± 5.2	41.6 ± 6.7	0.004
Mean arterial pressure (mmHg)	102.5 ± 7.6	90.4 ± 8.1	0.002
Heart rate (beats/min)	58.0 ± 8.2	65.4 ± 9.6	0.128
Stroke volume (mL)	112.6 ± 22.0	114.4 ± 17.6	0.687
Cardiac index (L/min/m^2^)	3.3 ± 0.6	3.6 ± 0.5	0.276
SVRI (dyne·sec/cm^5^/m^2^)	2392.2 ± 357.3	1865.3 ± 225.0	0.005
MFI	2.83 ± 0.15	2.91 ± 0.13	0.898
PPV (%)	94.4 ± 3.8	95.5 ± 3.3	0.734
FCD (mm/mm^2^)	19.0 ± 4.7^*∗*^	15.4 ± 2.9	0.041
TVD (mm/mm^2^)	20.1 ± 5.0^*∗*^	16.0 ± 2.7	0.040

Data are presented as mean ± SD. VO_2_max, maximum oxygen uptake; SVRI, systemic vascular resistance index; MFI, microvascular flow index of small vessels; PPV, percentage of perfused small vessels; TVD, total vessel density of small vessels; FCD, functional capillary density. ^*∗*^*p* < 0.05 compared with controls.

**Table 2:** Changes in microcirculatory parameters in marathon runners during the study period.

	Visit I	Visit II	Visit III	Visit IV	ANOVA, *p*
MFI	2.83 ± 0.15	2.92 ± 0.16	2.93 ± 0.12	2.89 ± 0.10	0.704
PPV (%)	94.4 ± 3.8	96.6 ± 2.0	96.7 ± 3.7	95.0 ± 3.6	0.409
FCD (mm/mm^2^)	19.0 ± 4.7^*∗*^	19.5 ± 4.1^*∗*^	18.6 ± 3.8^*∗*^	19.1 ± 4.3^*∗*^	0.976
TVD (mm/mm^2^)	20.1 ± 5.0^*∗*^	19.8 ± 5.3^*∗*^	19.8 ± 4.9^*∗*^	20.5 ± 5.1^*∗*^	0.989

Data are presented as mean ± SD. MFI: microvascular flow index of small vessels; PPV: percentage of perfused small vessels; TVD: total vessel density of small vessels; FCD: functional capillary density. Visit I, 24 hours before the marathon; Visit II, right after finishing the marathon; Visit III, 24 hours after the marathon; Visit IV, one week after the marathon. ^*∗*^*p* < 0.05 compared with controls.

**Table 3:** Subgroup analysis of marathon runners with increased or decreased FCD immediately after the marathon (Visit II) compared with Visit I.

	Decreased FCD (*n* = 6)	Increased FCD (*n* = 7)	*p*
Change in FCD	−1.96 ± 1.19	2.22 ± 2.86	0.004
Running duration (min)	190.37 ± 30.2	221.80 ± 23.4	0.045
Fluids during running (mL)	907 ± 615	1950 ± 488	0.007
Fluids after running (mL)	307 ± 341	100 ± 198	0.218
Change in mass (kg)	−2.4 ± 1.3	−1.0 ± 0.8	0.042
Change in MAP (mmHg)	−10.1 ± 9.0	−11.4 ± 7.6	0.806
Change in HR (beats/min)	22.6 ± 8.8	15.8 ± 7.6	0.196
Change in CO (L/min)	1.2 ± 1.2	1.1 ± 1.2	0.834
Change in SVRI (dyne·sec/cm^5^/m^2^)	−580.1 ± 323.8	−454.2 ± 221.3	0.471

Data are presented as mean ± SD. MAP: mean arterial pressure; HR: heart rate; CO: cardiac output; SVRI: systemic vascular resistance index.
